# Heterologous expression of a *Streptomyces cyaneus* laccase for biomass modification applications

**DOI:** 10.1186/s13568-017-0387-0

**Published:** 2017-04-24

**Authors:** Selin Ece, Camilla Lambertz, Rainer Fischer, Ulrich Commandeur

**Affiliations:** 1 Institute for Molecular Biotechnology (Biology VII), RWTH Aachen University, Worringerweg 1, 52074 Aachen, Germany; 2 Fraunhofer Institute for Molecular Biology and Applied Ecology (IME), Forckenbeckstrasse 6, 52074 Aachen, Germany

**Keywords:** Laccase, *Streptomyces cyaneus*, Heterologous expression, Enzyme immobilization, Lignocellulose modification

## Abstract

Laccases are used for the conversion of biomass into fermentable sugars but it is difficult to produce high yields of active laccases in heterologous expression systems. We overcame this challenge by expressing *Streptomyces cyaneus* CECT 3335 laccase in *Escherichia coli* (*Sc*Lac) and we achieved a yield of up to 104 mg L^−1^ following purification by one-step affinity chromatography. Stability and activity assays using simple lignin model substrates showed that the purified enzyme preparation was active over a broad pH range and at high temperatures, suggesting it would be suitable for biomass degradation. The reusability of *Sc*Lac was also demonstrated by immobilizing the enzyme on agarose beads with a binding yield of 33%, and by the synthesis of cross-linked enzyme aggregates with an initial activity recovery of 72%.

**Electronic supplementary material:**

The online version of this article (doi:10.1186/s13568-017-0387-0) contains supplementary material, which is available to authorized users.

## Introduction

Laccases are the largest subgroup of the multi-copper oxidase protein superfamily (Ihssen et al. 2015). They can oxidize a broad range of substrates including phenolic compounds, azo dyes, aromatic amines, non-phenolic substrates (mostly with the help of mediators), anilines and aromatic thiols, and recalcitrant environmental pollutants (Canas and Camarero 2010; Majumdar et al. 2014; Margot et al. 2013; Widsten and Kandelbauer 2008). Each monomeric laccase contains four copper atoms located at three different positions, namely the type 1 (T1), type 2 (T2) and binuclear type 3 (T3) copper sites, all of which are involved in the oxidation of substrate molecules accompanied by the reduction of molecular oxygen to two molecules of water (Thurston 1994). The copper atoms bind histidine residues that are conserved among the laccases of different organisms (Claus 2003; Luis et al. 2004). The T1 copper gives the laccase its blue color and is also responsible for the final oxidation of the substrate. Electrons are transferred from the T1 copper site to the T2/T3 sites, where molecular oxygen is reduced to water. The T1 copper is characterized by its absorbance at 610 nm whereas the T3 copper shows weak absorbance at 330 nm. The T2 copper is colorless but it can be detected by electro-paramagnetic resonance spectroscopy (EPR) (Gunne et al. 2013; Thurston 1994).

Laccases are used in many biotechnological processes in the paper and pulp, textile, pharmaceutical and petrochemical industries, and also for the bioremediation of industrial wastes (Chandra and Chowdhary 2015; Morozova et al. 2007; Munk et al. 2015; Rodriguez Couto and Toca Herrera 2006; Roth and Spiess 2015). Laccases can also be combined with laccase mediator systems (LMS) such as 1-hydroxybenzotriazole (HBT) for the pretreatment and depolymerization of lignocellulosic biomass (Call and Mücke 1997). The digestibility of cellulose can be increased following lignin decomposition by laccases (Chen et al. 2012). In the presence of HBT, lignin can also be removed from whole woody and non-woody feedstocks to increase sugar and ethanol yields (Gutierrez et al. 2012), whereas the alternative mediator 2,2′-azino-bis (3-ethylbenzothiazoline-6-sulfonic acid) (ABTS) can allow an alkaline-stable laccase to selectively degrade lignin within lignocellulose and improve the enzymatic hydrolysis of wheat straw when combined with a steam explosion pretreatment (Qiu and Chen 2012).

Although laccases are ubiquitous, research has focused mainly on fungal laccases because many different isozymes have been identified, particularly among the white-rot fungi. Following the identification of the first bacterial laccase (Givaudan et al. 1993) many further examples were discovered (Chandra and Chowdhary 2015). The properties of bacterial laccases, such as their enantioselectivity and stability at high pH and high temperatures, are not yet understood in detail, but they have many advantages for applications such as the pretreatment of recalcitrant biomass. The large-scale production of fungal laccases is challenging because of the slow growth rates of fungi. They also have a narrower optimal pH range. These factors have made bacterial laccases a valuable alternative (Ausec et al. 2011; Bugg et al. 2011; Chandra and Chowdhary 2015).

Here we describe the heterologous expression of a laccase from *Streptomyces cyaneus* CECT 3335 in *Escherichia coli* (*Sc*Lac). After purification, the recombinant enzyme preparation was characterized and compared in terms of its activity against common substrates. Two immobilization methods were used to assess the reusability of the recombinant laccase, thus offering a way to reduce the costs of enzyme production.

## Materials and methods

### Cloning

The laccase coding sequence (GenBank HQ857207) was codon optimized for expression in *E. coli* (European Nucleotide Archive LT795002), synthesized by Genscript (Piscataway, USA) and transferred to the expression vector pET-22b(+) (Novagen, Darmstadt, Germany). The gene was inserted at the *Nde*I and *Xho*I sites using forward primer 5′-GGA ATT CCA TAT GGA AAC CGA TAT TAT TGA ACG CC-3′ and reverse primer 5′-AAG CTC GAG GCC GGT ATG GCC CGC GCC ATG-3′. A His_6_ tag was added to the C-terminus to enable protein purification by immobilized metal affinity chromatography (IMAC). *E. coli* BL21 (DE3) Star (Novagen, Merck KGaA, Darmstadt, Germany) was used as the expression host.

### Expression and protein extraction

Transformed *E. coli* BL21 (DE3) Star cells were incubated overnight at 37 °C shaking at 180 rpm in 50 mL lysogeny broth (LB) containing 100 µg mL^−1^ ampicillin. The overnight culture was then used to inoculate 500 mL terrific broth (TB) supplemented with the same antibiotics, and the culture was incubated as described above until the optical density (OD_600_) reached 0.6. Laccase expression was then induced by adding 0.04 mM isopropyl β-d-1-thiogalactopyranoside (IPTG). Furthermore, 10 mM benzyl alcohol was added to the culture 20 min before the IPTG to induce the expression of native chaperones (de Marco et al. 2005). The laccase culture was incubated at 20 °C for 20 h at 180 rpm and the cells were harvested by centrifugation (5000×*g*, 15 min, 4 °C). The cell pellet was resuspended in 20 mM potassium phosphate buffer (pH 7.4) containing 20 mM imidazole and 300 mM NaCl. The cells were disrupted by sonication in the presence of 0.5 mM phenylmethylsulfonyl fluoride, 1 mg mL^−1^ lysozyme and 10 µg mL^−1^ DNase I. The cell suspension was then centrifuged (30,000×*g*, 30 min, 4 °C) and the supernatant was separated from the cell debris by passing through a 0.45-µm filter.

### Purification

*Sc*Lac was purified by IMAC using 5 mL HiTrap Chelating Sepharose FF (GE Healthcare, Freiburg, Germany). The cell extract was applied to the column at a flow rate of 3 mL min^−1^ in potassium phosphate running buffer (pH 7.4) containing 20 mM imidazole and 300 mM NaCl (also used as the washing buffer). Bound proteins were eluted by using a gradient of imidazole (0–500 mM) with a total volume of 50 mL. Elution fractions (2 mL) were checked by sodium dodecyl sulfate polyacrylamide gel electrophoresis (SDS-PAGE) using 5–12% Tris–glycine gels (Laemmli 1970). The gels were stained with Coomassie Brilliant Blue or blotted onto Hybond-C nitrocellulose membranes (GE Healthcare, Freiburg, Germany). The membranes were blocked with 5% (w/v) skimmed milk in phosphate buffered saline (PBS) for 1 h and incubated with a polyclonal antibody against the His_6_ tag (Rockland Immunochemicals, Limerick, USA) for at least 2 h at room temperature (RT) with constant shaking. After washing, the membranes were incubated with a polyclonal alkaline phosphatase-conjugated goat anti-rabbit (GAR^AP^) secondary antibody (Dianova, Hamburg, Germany) and the signal was visualized with nitroblue tetrazolium chloride/5-bromo-4-chloro-3-indolyl phosphate (NBT/BCIP) *p*-toluidine salt (Carl Roth, Karlsruhe, Germany). The protein bands were compared with P7712S molecular weight markers (New England Biolabs, Ipswich, USA). All fractions containing the target protein were pooled and dialyzed against 100 mM HEPES (pH 7.5) overnight at 4 °C with one buffer change. The purified and quantified proteins were stored at 4 °C.

Following dialysis, 0.5 mM CuSO_4_ was added to the laccase solution (Pozdnyakova and Wittung-Stafshede 2001) and the sample was mixed slowly on ice for 2 h before centrifugation (30,000×*g*, 30 min, 4 °C) to remove any aggregates that may have formed during the incubation with CuSO_4_. The protein concentration was determined again by measuring the absorbance at 280 nm and using the molar extinction coefficient of the *S. cyaneus* laccase coding sequence including the C-terminal His_6_ tag. The purified and quantified protein was stored briefly at 4 °C or was frozen in liquid nitrogen for long-term storage at −80 °C.

SDS-PAGE was used to analyze 13 µg of the purified *Sc*Lac preparation. The enzyme solution was separated on 5–12% Tris–glycine gels before staining with Coomassie Brilliant Blue, and the single protein band was compared to the P7712S molecular weight marker.

### Activity assays

Zymography assays were used for the initial verification of enzyme activity. Otherwise, the activity assays for *Sc*Lac were based on spectrophotometry, using an Infinite^®^ 200 microplate reader (Tecan, Maennedorf Switzerland) at 30 °C in 100 mM MES buffer (pH 5.5). Activity was tested by measuring the oxidation of the following substrates: 2,6-dimethoxyphenol (DMP; Sigma-Aldrich, Darmstadt, Germany) at 468 nm (ε = 49,600 M^−1^ cm^−1^), ABTS at 420 nm (ε = 36,000 M^−1^ cm^−1^) and guaiacol (Sigma-Aldrich, Darmstadt, Germany) at 465 nm (ε = 26,600 M^−1^ cm^−1^). Control reactions were prepared under the same conditions with combinations of purified laccase and buffer, substrate and buffer, or buffer only. All activity assays were performed either in duplicates or in triplicates.

### Characterization of the purified enzyme

The ultraviolet/visible (UV–Vis) spectra (230–700 nm) of 1 µM purified *Sc*Lac was recorded before and after incubation with CuSO_4_ using an Infinite 200 plate reader. The activity assay before and after the incubation with CuSO_4_ was carried out using DMP as the substrate with 0.4 µM *Sc*Lac.

The pH optimum of the laccase was determined by measuring the activity of the purified recombinant enzyme against DMP as described above, in a set of buffers with pH values ranging from 3.5 to 10.0. The buffers were 100 mM sodium acetate (pH 3.5–5.0), 100 mM 3-(*N*-morpholino)propanesulfonic acid (MOPS) (pH 5.5–7.5), 100 mM 2-amino-2-hydroxymethyl-propane-1,3-diol hydrochloric acid (Tris–HCl) (pH 8.0–8.5) and 100 mM glycine sodium hydroxide (pH 9.0–10.0). Each reaction was prepared with 0.4 µM *Sc*Lac and the reactions were followed at 468 nm for 15 min. Enzyme stability in the optimal buffer system was determined by incubating 0.4 µM *Sc*Lac for 1, 6 and 24 h at 30 °C before measuring residual activities against DMP as described above. Control reactions were set up with 0.4 µM laccase without incubation at 30 °C.

The temperature optimum of *Sc*Lac was determined by incubating 0.4 µM of the enzyme with 20 µM DMP in 100 mM MES buffer (pH 5.5) at various temperatures ranging from 25 to 90 °C for 5 min, and then measuring the absorbance values as described for DMP above. The influence of temperature on laccase stability was determined by incubating 0.4 µM *Sc*Lac in 100 mM MES buffer (pH 5.5) at 25, 30, 60 and 90 °C for 1 h, then chilling the protein samples on ice for 5 min and measuring the residual enzyme activity using DMP as described above. The diagrams for the characterization of *Sc*Lac were based on relative activities calculated by assigning the highest value in the dataset representing each enzyme as 100%.

Kinetic parameters were analyzed by measuring enzyme activities against guaiacol, DMP and ABTS (as described above) over the concentration range 5–95 µM under the optimal assay conditions. Kinetic constants were analyzed and calculated using GraphPad Prism v6 software (Statcon, Germany).

### Immobilization of *Sc*Lac

A sample of purified *Sc*Lac was immobilized using two different methods: cross linked enzyme aggregates (CLEAs) and AminoLink™ Plus Coupling Resin (Thermo Fisher Scientific, Darmstadt, Germany). The latter was used as a carrier material benefiting from the Schiff base reaction between the primary amines of the protein and the aldehyde groups of the resin.

CLEAs were prepared as follows: 2.5 mL of 1 g mL^−1^ polyethylene glycol (PEG) 4000 was added dropwise to 5 mg of a purified *Sc*Lac sample on ice and incubated at 20 °C for 2 h, shaking at 200 rpm. The sample was mixed with 5 mM glutaraldehyde and incubated overnight under the same conditions. The CLEAs were then collected by centrifugation (5000×*g*, 10 min, 4 °C). The supernatant was removed and an activity assay with DMP was carried out as described above to check for free laccase in the washing fraction. The washing steps were repeated until no activity was detected in the washing buffer and the CLEAs were then resuspended in 1 mL 0.1 M MES buffer (pH 5.5). The activity recovery after the CLEA protocol was calculated by dividing the CLEA activity (U) by the activity of the free enzyme (U L^−1^) multiplied by the volume of the free enzyme used for immobilization, and then multiplying the resulting value by 100 (Eq. 1) (Lopez-Gallego et al. 2005).

Calculation of the activity recovery (%) of prepared CLEAs. A_CLEA_:

1 $${\text{Activity recovery}} \,\left( \% \right) = \frac{{{\text{A}}_{\text{CLEA}} }}{{{\text{A}}_{\text{free}} \times {\text{V}}_{\text{free}} }} \times 100$$

where activity (U) of prepared CLEA; A_Free_: activity (U mL^−1^) of free enzyme; V_Free_: volume (mL) of free enzyme used to prepare CLEAs.

Immobilization on AminoLink™ Plus Coupling Resin was achieved by mixing approximately 5 mg of purified *Sc*Lac with 10–50 µm aldehyde-functionalized agarose beads provided as a 50% slurry in 0.02% sodium azide buffer. The immobilization protocol was performed in 10 mL gravity-flow columns (Bio-Rad, Munich, Germany) that were never allowed to run dry. The columns were loaded with 500 μL of the bead slurry before equilibration with 6 mL coupling buffer (0.1 M sodium phosphate, 0.15 M NaCl, pH 7.2). The columns were then loaded with 4.5 mL of the protein sample (1.11 mg mL^−1^) and the slurry was mixed for 3–4 h at 4 °C. The samples were then drained and the beads were equilibrated in 3 mL coupling buffer before adding 1 mL 0.1 M sodium cyanoborohydride in coupling buffer and mixing overnight at 4 °C. The reaction buffer was drained and the beads were equilibrated with 2 mL quenching buffer (1 M Tris–HCl, pH 7.4). Following the equilibration, 1 mL 0.1 M sodium cyanoborohydride in quenching buffer was added to the beads and the slurry was mixed for a further 90 min. To complete the immobilization, the beads were washed with 6 mL washing buffer and stored in 500 μL immobilization storage buffer.

The protein concentration on the beads was calculated by subtracting the mass of protein after the first reaction step and the mass of the protein lost during the washing steps from the initial mass of protein, and dividing this by the volume of the bead slurry after immobilization. Protein concentrations for immobilization experiments were determined using the bicinchoninic acid assay (Pierce™ BCA Protein Assay Kit, Thermo Fisher Scientific, Darmstadt, Germany) according to the manufacturer’s protocol. All samples and standards were measured either in duplicates or triplicates.

The reusability of the immobilized laccase preparations was assessed in five sequential activity assays against DMP. Immediately prior to the reusability assay, the enzyme samples were mixed vigorously. After each activity assay, beads and CLEAs were collected by either gravity or centrifugation (15,000×*g*, 5 min, 4 °C) and washed three times. Temperature stability and pH optima were determined as described above. The diagrams for the characterization of immobilized *Sc*Lac preparations were based on relative activities calculated by assigning the highest value in each dataset as 100% (except for the reusability assay). For the reusability assay, the first measurement in each dataset was set to 100%.

## Results

### Heterologous expression of *S. cyaneus* CECT 3335 laccase in *E. coli* and purification by IMAC

**Fig. 1:**
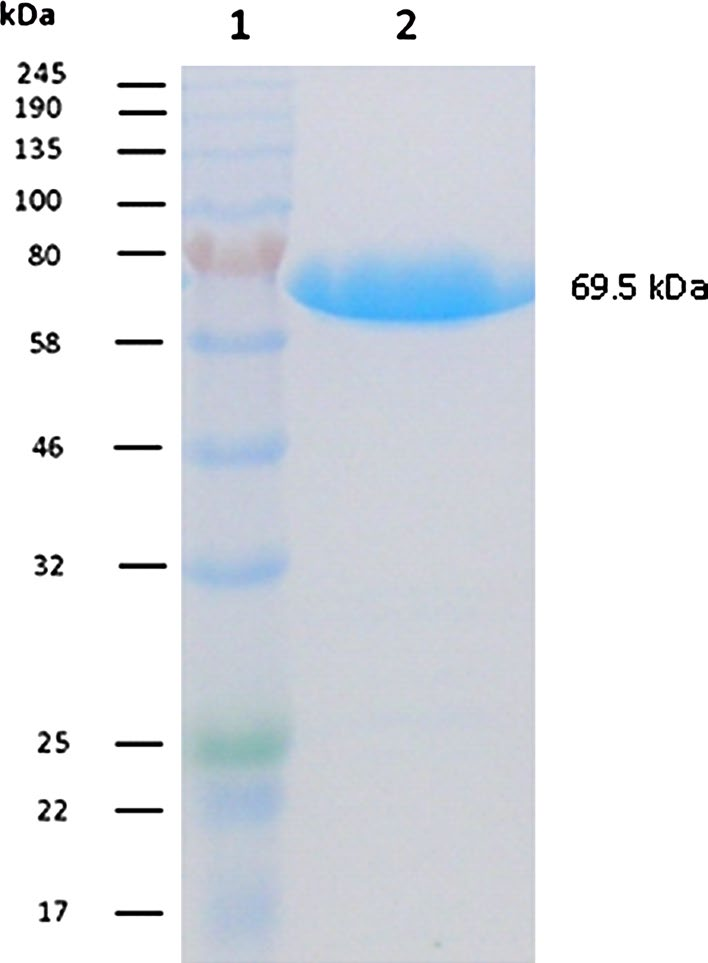
SDS-PAGE analysis of purified laccase expressed in *E. coli*. *Lane 2* was loaded with 13 µg of protein and the gel was stained with Coomassie Brilliant Blue G250. *1* Molecular mass marker. *2* IMAC-purified *Sc*Lac sample

*Streptomyces cyaneus* CECT 3335 laccase with a C-terminal His_6_ tag was expressed successfully in *E. coli*. After purification by single-step IMAC, SDS-PAGE analysis revealed a strong band for the enzyme preparation near the 70 kDa marker agreeing with the predicted molecular mass of 69.5 kDa (Fig. 1). High yields (up to 104 mg L^−1^) of purified recombinant *Sc*Lac were achieved using *E. coli*, suggesting that despite its simplicity, it remains a promising host organism for the production of substantial amounts of this enzyme for further biotechnological applications.

### Analysis of the enzymatic activity and functional properties of recombinant *Sc*Lac

**Fig. 2:**
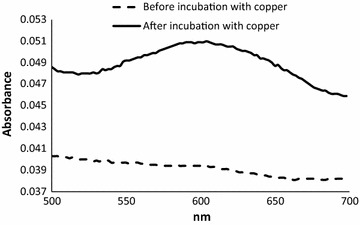
Visible spectra of *Sc*Lac before (*dashed line*) and after (*solid line*) incubation with 0.5 mM CuSO_4_. We used 1 µM of recombinant enzyme to record the visible spectra between 500 and 700 nm

The visible spectra of the purified *Sc*Lac correlated with the typical spectra of blue laccases. A peak at ~600 nm, which was detected only after incubation with CuSO_4_, indicated that the T1 copper atom was incorporated into the protein structure (Thurston 1994) (Fig. 2). Activity assays using DMP before and after incubation with CuSO_4_ confirmed that the laccase was expressed as an apoprotein in *E. coli* and the addition of copper was necessary for the maturation and activation of the enzyme (Additional file 1: Fig. S1). Zymography assays using ABTS, l-DOPA and caffeic acid as substrates (Additional file 1: Fig. S2) also confirmed laccase activity following incubation with CuSO_4_.

**Fig. 3:**
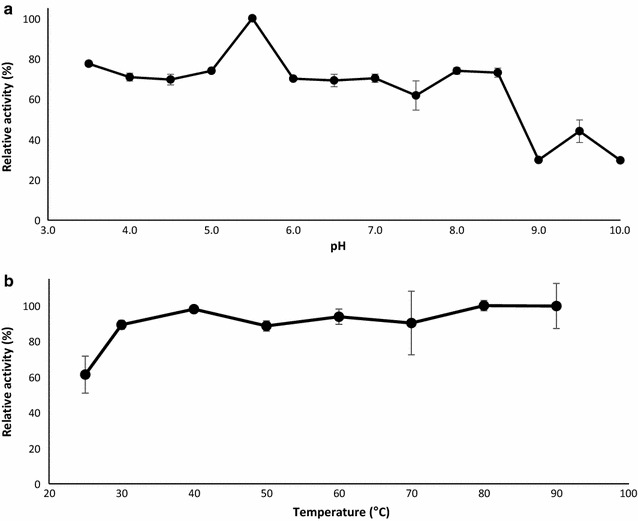
Optimum pH (**a**) and temperature (**b**) profiles of *Sc*Lac. **a** The activity of the laccase was measured against 20 µM DMP at 30 °C in buffers with various pH values ranging from pH 3.5–10.0. **b** The activity of the laccase was measured against 20 µM DMP after incubation for 5 min in the optimal buffer system in a temperature range from 25 to 90 °C

**Fig. 4:**
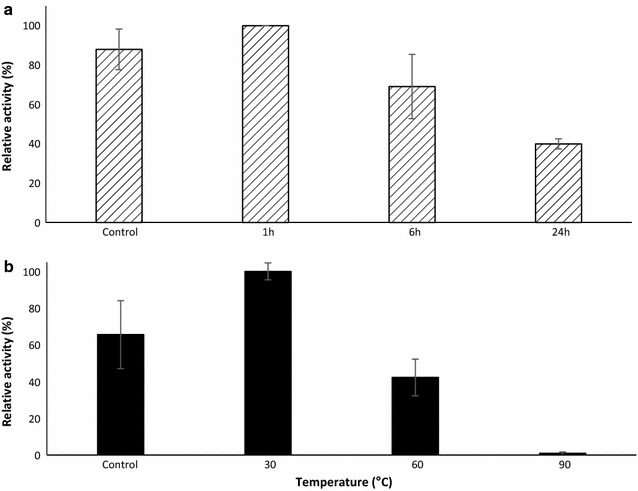
The pH (**a**) and thermal (**b**) stability profiles of *Sc*Lac. **a** We incubated 0.4 µM *Sc*Lac in the optimal pH buffer for 1, 6 and 24 h at 30 °C and the residual activity was measured using 20 µM DMP. **b** We incubated 0.4 µM *Sc*Lac at 30, 60 and 90 °C for 1 h in the optimal pH buffer and the residual activities were tested against 20 µM DMP. Control assays were set up without pH or heat treatment

The pH activity profile of the purified laccase was determined using DMP as the substrate with a set of buffers covering a broad pH range (pH 3.5–10.0). The recombinant *Sc*Lac reached its maximum activity at pH 5.5 (Fig. 3a), and was active across a broad range of pH values (pH 3.5–8.5). The pH stability of *Sc*Lac was investigated by incubating an aliquot of the purified enzyme preparation in the same buffer set used to determine the pH optima for 0, 1, 6 and 24 h (Fig. 4a). *Sc*Lac generally lost activity over time, but the decline was more rapid at pH 3.5–5.5 than at pH values >6. Although *Sc*Lac had a pH optimum of 5.5, the stability profile suggested that the enzyme is also active at neutral and basic pH values.

*Sc*Lac showed high activity at elevated temperatures, as previously described for other bacterial laccases and laccase-like multi-copper oxidases (LMCOs) (Ihssen et al. 2015; Koschorreck et al. 2008; Martins et al. 2002; Reiss et al. 2011; Sherif et al. 2013). It also showed a broad optimum temperature range of 30–90 °C (Fig. 3b). The heat stability of the recombinant enzyme was also determined during longer incubation periods. *Sc*Lac was incubated at 30, 60 and 90 °C for 1 h and subsequently tested for the remaining activity against DMP (Fig. 4b). The activity of the recombinant laccase increased during the incubation at 30 °C and it retained 50% of its initial activity following incubation at 60 °C but lost most of its activity at 90 °C.

### Determination of substrate specificity and steady-state kinetics

The specific activities and kinetic constants were determined using three common laccase substrates (DMP, ABTS and guaiacol) that are also used to test other lignin-degrading enzymes such as peroxidases (Table 1). The kinetic parameters for this particular laccase are presented here for the first time and fit within the range of values reported for other recombinant bacterial laccases (Dwivedi et al. 2011; Ihssen et al. 2015).

**Table 1:** Specific activities and kinetic constants of *Sc*Lac against DMP, ABTS and guaiacol

	DMP	ABTS	Guaiacol
Specific activity (U mg^−1^)	0.540 ± 0.020	2.9400 ± 0.1700	0.0190 ± 0.0010
Km (mM)	0.2016 ± 0.0348	0.0335 ± 0.0279	0.0054 ± 0.0019
Vmax (U)	0.0053 ± 0.0007	0.0003 ± 0.0002	0.0002 ± 0.0000
kcat (min^−1^)	57.30 ± 8.10	3.4640 ± 1.8640	1.6740 ± 0.0876

### Immobilization of *Sc*Lac

**Fig. 5:**
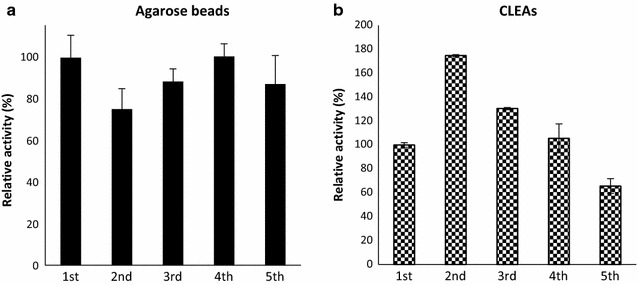
The reusability of immobilized *Sc*Lac. The remaining activity of immobilized *Sc*Lac on agarose beads (**a**) or as CLEAs (**b**) was analyzed in sequential activity assays for five cycles using DMP as the substrate

*Sc*Lac was immobilized on agarose beads and by the preparation of CLEAs, the latter mediated by PEG precipitation and glutaraldehyde cross-linking. At the end of the CLEA procedure, 71.5% of the initial activity was recovered. The immobilization of *Sc*Lac on agarose beads using a Schiff base reaction achieved 33% immobilization efficiency (the proportion of bound protein). The reusability of the immobilized enzyme preparations was tested by performing five sequential activity assays using DMP as the substrate. The laccase immobilized on agarose beads maintained its activity in all five steps. The activity of the CLEAs declined to ~60% of the initial activity over the five steps, but the heat activation detected in the free enzyme sample was also observed in the CLEA preparation (Fig. 5).

**Fig. 6:**
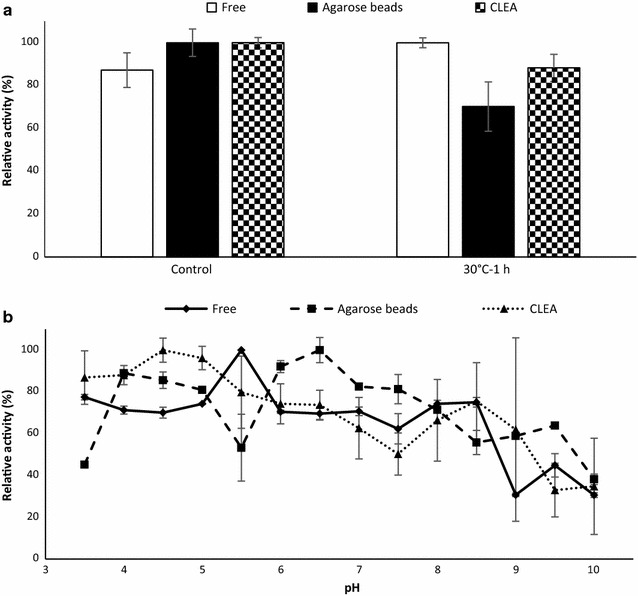
Characterization of immobilized and free *Sc*Lac. **a** Thermal stability was determined by incubating the free and immobilized enzymes at 30 °C for 1 h and measuring the residual activities. Control assays were set up without heat treatment. **b** Optimum pH profiles of free *Sc*Lac, *Sc*Lac immobilized on agarose beads and *Sc*Lac CLEAs

The stability of the immobilized *Sc*Lac was tested after 1 h incubation at 30 °C and compared to the free laccase. The laccase immobilized on agarose beads retained 70% of its initial activity whereas the CLEAs retained 88% of their initial activity (Fig. 6a). The pH optima of the immobilized enzymes were also determined and were found to differ from the free enzyme preparation most likely due to changes in the structural conformation of the enzyme or the microenvironment induced by the immobilization method or the matrix (Bussamara et al. 2012; Guzik et al. 2014; Kumar et al. 2014). The laccase immobilized on agarose beads reached maximum activity at pH 6.5 and the CLEAs reached maximum activity at pH 4.5 (Fig. 6b). Although the immobilized *Sc*Lac can be reused at least five times, neither the stability nor the activity of the immobilized enzyme improved compared to the free enzyme.

## Discussion

Laccases are found in all three domains of life and catalyze biotechnologically significant reactions. The characterization of bacterial laccases has shown that they have unique properties and are more advantageous than laccases from fungi and plants. However, the yield of purified bacterial laccases is a limiting factor for biotechnological applications. Here we expressed a laccase from the lignocellulose-mineralizing bacterium *S. cyaneus* in *E. coli* for the first time, achieving high yields of the soluble recombinant enzyme (104 mg L^−1^). The extracellular expression of *Sc*Lac in its native host was previously carried out for 10 days in a submerged culture, yielding 8.19 mg protein from 100 mL of culture supernatant following five purification steps (Arias et al. 2003; Margot et al. 2013). In contrast, the heterologous expression of *Sc*Lac in *E. coli* achieved higher yields in a shorter cultivation time and required only a single affinity chromatography purification step.

Several recombinant bacterial laccases have been expressed in *E. coli*, but in most cases the yields were poor (Table 2). Whereas low yields in homologous systems may be caused by the need for submerged cultures, heterologous systems face challenges such as codon usage bias, the need for signal peptides, protein accumulation as inclusion bodies, the absence of necessary post-translational modifications, and enzyme inactivation during purification (Brijwani et al. 2010; Piscitelli et al. 2010). The heterologous expression of CotA laccase from *Bacillus subtilis* in *E. coli* has been achieved by several groups and has led to detailed characterization studies, structural and functional analysis, and the use of protein evolution to improve the yields of recombinant enzyme (Bento et al. 2005; Brissos et al. 2009; Martins et al. 2002; Osipov et al. 2015). A blue multi-copper oxidase from *Marinomonas mediterranea* (PpoA) was expressed in *E. coli* BL21 (DE3) and laccase activity was observed against the substrates l-3,4-dihydroxyphenylalanine (l-DOPA), DMP and syringaldazine (SGZ) in the soluble fractions of the cell extracts (Sanchez-Amat et al. 2001). However, the yield of the recombinant enzyme was not reported and the purification strategy was not described. A laccase-like phenol oxidase from *Streptomyces griseus* (EpoA) was expressed in *E. coli* with a C-terminal His_6_ tag for purification by affinity chromatography and ion exchange chromatography (Endo et al. 2003). A thermostable laccase from *Streptomyces lavendual*e was expressed in *E. coli* with a yield of up to 30 mg L^−1^ and 10 mg of pure protein was isolated after five purification steps (Suzuki et al. 2003).

**Table 2:** Bacterial laccases, laccase-like phenol oxidases and multi-copper oxidases produced by heterologous expression in *E. coli*

Enzyme	Origin	Yield	Comments	Reference
PpoA	*Marinomonas mediterranea*	NR		Sanchez-Amat et al. (2001)
Cot A	*Bacillus subtilis*	NR	10% of total recombinant CotA expressed by *E. coli* was purified successfully	Martins et al. (2002)
CotA	*B. subtilis*	20 mg L^−1^ (aerobic) 23 mg L^−1^ (anaerobic)		Durao et al. (2006, 2008a, b); Pereira et al. (2009)
EpoA	*Streptomyces griseus*	NR		Endo et al. (2003)
STSL	*Streptomyces lavendulae*	10 mg L^−1^		Suzuki et al. (2003)
Lbh1	*Bacillus halodurans*	NR		Ruijssenaars and Hartmans (2004)
SLAC	*Streptomyces coelicolor*	NR		Machczynski et al. (2004)
Tth laccase	*Thermus thermophilus*	10 mg L^−1^		Miyazaki (2005)
McoA	*Aquifex aeolicus*	NR	Expressed as insoluble protein and recovered by unfolding and refolding	Fernandes et al. (2007)
CotA	*Bacillus licheniformis*	10 mg L^−1^		Koschorreck et al. (2008)
SilA	*Streptomyces ipomea*	NR	4.8-fold purification of the protein corresponding to a final yield of 85.6%	Molina-Guijarro et al. (2009)
LCMOs	*Bacillus coagulans* *Bacillus clausii* *Bacillus pumilus* *Streptomyces pristinaespiralis* *Gramella forsetii* *Marivirga tractuosa* *Spirosoma linguale*	NR		Ihssen et al. (2015)
SCLAC SLLAC SVLAC AMLAC	*S. coelicolor* *Streptomyces lividans* *Streptomyces viridosporus* *Amycolatopsis* sp.	15–20 mg L^−1^		Majumdar et al. (2014)
Cot A	*B. clausii*	NR		Brander et al. (2014)
Ssl1	*Streptomyces sviceus*	40–50 mg L^−1^		Gunne and Urlacher (2012)
BALL	*B. licheniformis*	20 mg L^−1^		Tonin et al. (2016)
MmPPO	*Marinomonas mediterranea*	NR	Recovered from membrane fraction and partially purified to 34 U L^−1^	Tonin et al. (2016)

The enzyme yield is the amount of enzyme recovered after purification

*NR* not reported

Other laccases and LMCOs have been expressed in *E. coli* (Table 2) including the Lbh1 multi-copper oxidase from *Bacillus halodurans*, which has alkaline laccase activity (Ruijssenaars and Hartmans 2004), a small laccase from *Streptomyces coelicolor* (SLAC) (Machczynski et al. 2004), the hyperthermophilic Tth laccase from *Thermus thermophilus* (Miyazaki 2005), a robust metallo-oxidase (McoA) from the hyperthermophilic bacterium *Aquifex aeolicus* (Fernandes et al. 2007), CotA laccases from *Bacillus* spp. (Brander et al. 2014; Guan et al. 2014; Koschorreck et al. 2008), and a pH-versatile, salt-resistant laccase from *Streptomyces ipomoea* (SilA) (Molina-Guijarro et al. 2009). Although the heterologous expression of these bacterial laccases in *E. coli* made it possible to characterize the recombinant laccases and perform detailed structural analysis, the yields were often low or the proteins formed inclusion bodies, which made purification more laborious. In contrast, we achieved the production of substantial amounts of a recombinant laccase for further applications.

UV/Vis spectra of the recombinant *Sc*Lac preparation and subsequent activity assays showed that the T1 copper was not incorporated into the recombinant protein structure during its expression in *E. coli*. A similar phenomenon was reported for recombinant EpoA, which was only active when expressed in the presence of 10 µM CuSO_4_. Otherwise, the enzyme accumulated in an inactive monomeric form (Endo et al. 2003). However, the addition of copper to the expression medium might not be advisable because excess copper is toxic and may inhibit cell growth (Bird et al. 2013; Grey and Steck 2001). The incorporation of the T1 copper into *Sc*Lac was achieved by incubating the purified enzyme preparation in a buffer system containing 0.5 mM copper, and confirmed by UV–Vis spectra and subsequent activity assays. Unlike T1 copper, the verification of T2 and T3 copper incorporation requires other techniques such as EPR rather than UV–Vis spectra. After incubation with CuSO_4_, enzymatic activity tests by zymography revealed laccase activity against ABTS, l-DOPA and caffeic acid. Further analysis of specific activities and kinetics showed that the recombinant *Sc*Lac was most active against ABTS followed by DMP and guaiacol, similar to other bacterial laccases.

The recombinant *Sc*Lac showed broad optimum pH and temperature ranges. *Sc*Lac showed its maximum activity at pH 5.5 and it retained its stability at neutral and basic pH values, in agreement with reports describing other bacterial laccases. This leads to the suggestion that *Sc*Lac also might be ideal for biotechnological applications where neutral or basic pH values are required over a longer period (Brander et al. 2014; Gunne and Urlacher 2012; Ruijssenaars and Hartmans 2004). An activity at neutral or basic pH values is often observed for bacterial laccases and laccase-like enzymes but less frequently for the fungal laccases (Christopher et al. 2014). For example, *B. halodurans* Lbh1 showed maximum activity against SGZ at pH 7.5–8.0, *Streptomyces sviceus* Ssl1 showed maximum activity against phenolic substrates such as DMP and guaiacol at pH 9.0 and against SGZ at pH 8.0, and a halotolerant alkaline laccase from *Streptomyces psammoticus* showed maximum activity at pH 8.5 and retained 97% of its initial activity after 90 min at pH 9.0 (Gunne and Urlacher 2012; Niladevi et al. 2008; Ruijssenaars and Hartmans 2004). High activities in alkaline solutions are ideal for industrial applications such as the bio-bleaching of Kraft pulp during paper production, lignin modification and total biomass degradation (Pometto and Crawford 1986; Ruijssenaars and Hartmans 2004; Si et al. 2015). Laccases often remain active for a short time at high temperatures (Reiss et al. 2011; Zhang et al. 2013) but industrial applications usually require prolonged reactions. Although *Sc*Lac lost most of its activity following the incubation at 90 °C, the stability profiles at 30 and 60 °C were promising. A phenomenon known as heat activation, which has already been reported for a *Bacillus clausii* LMCO expressed in *E. coli* (Brander et al. 2014), was observed for *Sc*Lac after incubation at 30 °C.

The cost of enzyme production is a major factor in the economics of enzyme-based biomass degradation processes and is dependent on the host cells and purification strategy (Klein-Marcuschamer et al. 2012). Such costs can be minimized if the enzyme is reused in multiple process cycles by means of immobilization. In this study, *Sc*Lac was immobilized using two distinct methods: agarose beads and cross-linked enzyme aggregates (CLEA). Unlike reported for several laccases (Cabana et al. 2007; Sinirlioğlu et al. 2013), the stability of immobilized *Sc*Lac was not improved in comparison to the free enzyme. However, the recovery and reusability of the enzyme was demonstrated successfully, suggesting that *Sc*Lac could be used for the development of cost-efficient biotechnological processes (Robinson 2015).

## Additional file

**Additional file 1.** Additional figures.

## Abbreviations

ABTS: 2,2′-azinobis(3-ethylbenzothiazoline-6-sulfonate)
CLEA: cross-linked enzyme aggregate
DMP: 2,6-dimethoxyphenol
IMAC: immobilized metal affinity chromatography
l-DOPA: l-3,4-dihydroxyphenylalanine
LMCO: laccase-like multi-copper oxidase
LMS: laccase mediator system
MOPS: 3-(*N*-morpholino) propanesulfonic acid
PBS: phosphate-buffered saline
PEG: polyethylene glycol
PMSF: phenylmethylsulfonyl fluoride
RT: room temperature
*Sc*Lac: laccase from *Streptomyces cyaneus* expressed in *E. coli*
SDS-PAGE: sodium dodecylsulfate polyacrylamide gel electrophoresis
SGZ: syringaldezine(4-hydroxy-3,5-dimethoxybenzaldehyde azine)
VA: veratryl alcohol
