# Supplementary material for: Heterologous expression of a Streptomyces cyaneus laccase for biomass modification applications
Source: AMB Express. 2017 Apr 24;7:86. doi: 10.1186/s13568-017-0387-0 (PMC5403781; doi:10.1186/s13568-017-0387-0)
Supplement: Supplementary file 1 — Additional file 1. Additional figures. [file 13568_2017_387_MOESM1_ESM.pdf]

Title: Heterologous expression of a *Streptomyces cyaneus* laccase for biomass modification applications

Supplementary Material

AMB Express

Authors: Selin Ece[1], Camilla Lambertz[1], Rainer Fischer[1,2] and Ulrich Commandeur[1]*

Affiliations:

[1] Institute for Molecular Biotechnology (Biology VII), RWTH Aachen University, Worringerweg 1, Aachen, 52074, Germany

[2] Fraunhofer Institute for Molecular Biology and Applied Ecology (IME), Forckenbeckstrasse 6, Aachen, 52074, Germany

* Corresponding author: Institute for Molecular Biotechnology (Biology VII), RWTH Aachen University, Worringerweg 1, Aachen, 52074, Germany

E-mail: ulrich.commandeur@molbiotech.rwth-aachen.de

Phone: +49-241-80-28131

Fig. S1 Activity assays of *Sc*Lac before and after incubation with 0.5 mM $CuSO_4$.

Fig. S2 Zymogram analysis of purified laccase expressed in *E. coli*.

Supplementary figures

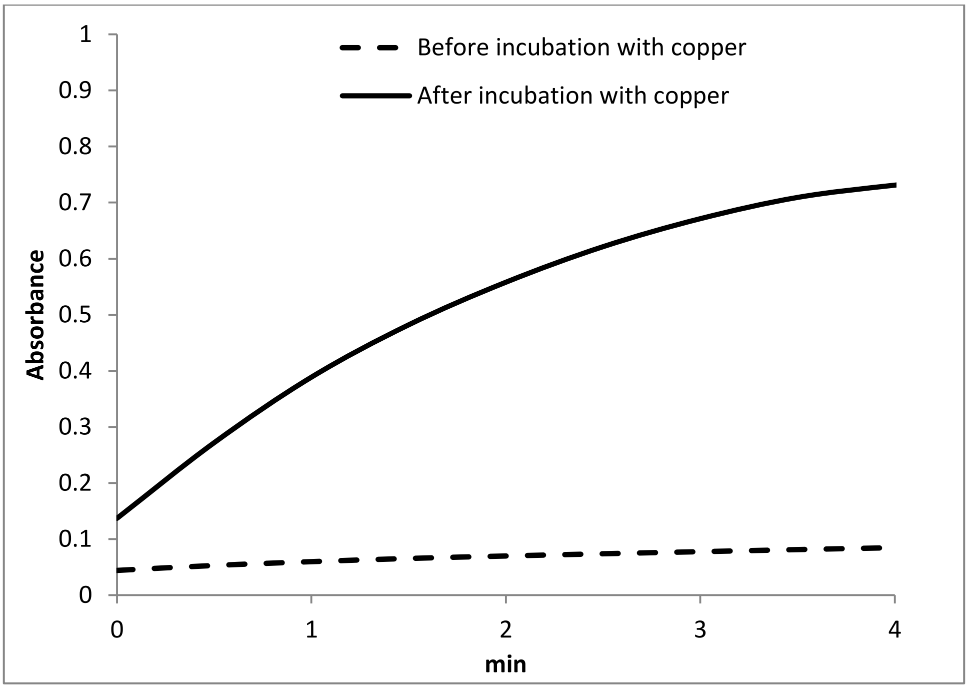

Fig. S1 Activity assays of *Sc*Lac before (dashed line) and after (solid line) incubation with 0.5 mM $CuSO_4$. We used 0.4 µM *Sc*Lac in the assays with DMP as the substrate. The reactions were followed for 4 min at 468 nm

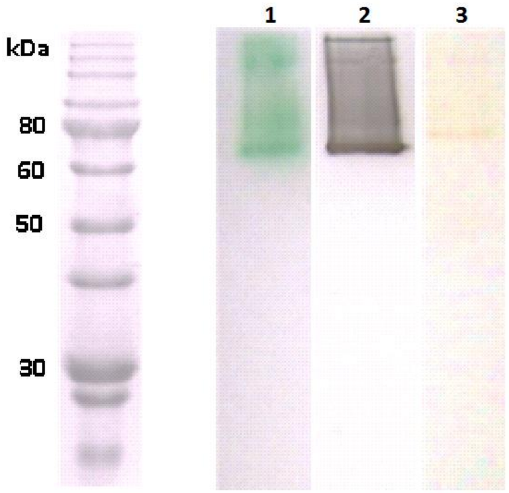

Fig. S2 Zymogram analysis of purified laccase expressed in *E. coli*. Native protein gel electrophoresis was carried out using 5–12% native-PAGE Tris-glycine gels loaded with 25 µg of laccase per lane. When the separation was completed, the gels were soaked in 10 mM ABTS (1), L-DOPA (2) or caffeic acid (3) prepared in 100 mM MES buffer (pH 5.5) and incubated at 37°C for 5 min. Reactions were stopped by exchanging the buffer with 1:1 ethanol-water (Ramachandra et al. 1987; Thomas et al. 1998). Molecular mass markers are shown on the left

References

Ramachandra M, Crawford DL, Pometto AL (1987) Extracellular enzyme-activities during lignocellulose degradation by *Streptomyces* Spp - A comparative-study of Wild-Type and genetically manipulated strains. Appl Environ Microbiol 53(12):2754-2760

Thomas M, Barker G, Furness PN (1998) A semi-quantitative approach to in situ zymography using tissue sections. J Pathol 186:4a-4a
